# Supplementary material for: Virus-Host Dynamics in Archaeal Groundwater Biofilms and the Associated Bacterial Community Composition
Source: Viruses. 2023 Mar 31;15(4):910. doi: 10.3390/v15040910 (PMC10143303; doi:10.3390/v15040910)
Supplement: Supplementary file 1 [file viruses-15-00910-s001.zip › Supplementary_Turzynski_et_al_2023/Supplementary_Material_Turzynski.docx]

Supplementary Information for:

Virus-host dynamics in archaeal groundwater biofilms and the associated bacterial community composition

Victoria Turzynski^1,2^, Lea Griesdorn^1,2^, Cristina Moraru^3^, André Soares^1,2^, Sophie A. Simon^1,2^, Tom L. Stach^1,2^, Janina Rahlff^1,†^, Sarah P. Esser^1,2^, and Alexander J. Probst^1,2,4,5#^

^1^Environmental Microbiology and Biotechnology (EMB), Department of Chemistry, Group for Aquatic Microbial Ecology, University of Duisburg-Essen, Universitätsstraße 5, 45141 Essen, Germany

^2^Environmental Metagenomics, Research Center One Health Ruhr of the University Alliance Ruhr, Faculty of Chemistry, University of Duisburg-Essen, Universitätsstraße 5, 45141 Essen, Germany

^3^Institute for Chemistry and Biology of the Marine Environment (ICBM), Carl-von-Ossietzky-University Oldenburg, PO Box 2503, Carl-von-Ossietzky-Straße 9-11, 26111, Oldenburg, Germany

^4^Centre of Water and Environmental Research (ZWU), University of Duisburg-Essen, Universitätsstraße 5, 45141 Essen, Germany

^5^Centre for Medical Biotechnology (ZMB), University of Duisburg-Essen, Universitätsstraße 5, 45141 Essen, Germany

^†^Present address: Centre for Ecology and Evolution in Microbial Model Systems (EEMiS), Department of Biology and Environmental Science, Linnaeus University, SE-39182, Kalmar, Sweden

^#^Corresponding author: alexander.probst@uni-due.de, telephone: +49 201 183-7080

Content:

1. Supplementary Tables
2. Supplementary Figures
3. Supplementary References

Supplementary Tables are provided as Supplementary_Tables.xlsx file, which contains the following individual sheets:

- **Table S1:** Sampling events on the Mühlbacher Schwefelquelle (MSI).
- **Table S2:** Viral enumeration of different infection stages with Altivir_1_MSI (results are visualized in Figure 1A).
- **Table S3:** Direct-geneFISH probes for targeting the genome of *Ca.* A. hamiconexum for the determination of the detection efficiency of virusFISH.
- **Table S4:** Melting profiles of the direct-geneFISH probes for targeting the genome of *Ca.* A. hamiconexum for the determination of the detection efficiency of virusFISH
- **Table S5:** Determining the detection efficiency of virusFISH (raw data).
- **Table S6:** Calculation of the detection efficiency of virusFISH (results are displayed in Fig. 2).
- **Table S7:** Melting profile of the primer set Altivir_1_MSI_F and Altivir_1_MSI_R for targeting the lytic virus Altivir_1_MSI in individual biofilm flocks from the MSI.
- **Table S8:** qPCR standard curves of the respective primer sets for targeting *Ca.* Altiarchaeum hamiconexum, Altivir_1_MSI and bacteria/archaea in individual MSI biofilm flocks.
- **Table S9:** Kruskal-Wallis and Dunn’s significance tests for qPCR and virusFISH data sets.
- **Table S10:** Nanopore sequencing - Barcode sequences for the16S Barcoding Kit.
- **Table S11:** Raw data of virus-host ratios of different methods, real-time PCR, metagenomics, and virusFISH (results are illustrated in Fig. 1B).
- **Table S12:** Targeting *Ca.* A. hamiconexum, its virus Altivir_1_MSI and the entire bacteriome within individual MSI biofilms by using real-time PCR (results are illustrated in Fig. 3).

**Figure S1:** VirusFISH on MSI biofilms by using a non-matching *Metallosphaera* sp. virus probe as a negative control for **Main Figure 3**.

**Figure S2:** Direct-geneFISH of *E. coli* cells as a negative control for **Main Figure 2C** with all 33 different polynucleotides that specifically target the *Ca.* Altiarchaeum genome.

**Figure S3:** Extended data of **Main Figure 2C**, methods according to main manuscript. Direct-geneFISH on MSI biofilms using eleven different polynucleotides to target the *Ca.* Altiarchaeum genome. Only strong, punctual signals were counted for calculating the labelling efficiency.

**Figure S4:** Extended data of **Main Figure 2C**, methods according to main manuscript. Direct-geneFISH on MSI biofilms using 22 different polynucleotides to target the *Ca.* Altiarchaeum genome. Only strong, punctual signals were counted for calculating the labelling efficiency.

**Figure S5:** Extended data of **Main Figure 2C**, methods according to main manuscript. Direct-geneFISH on MSI biofilms using 33 different polynucleotides to target the *Ca.* Altiarchaeum genome. Only strong, punctual signals were counted for calculating the labelling efficiency.

**Figure S6:** Extended data of **Main Figure 3A**, methods according to main manuscript. VirusFISH on MSI biofilms shows few viral infections caused by Altivir_1_MSI.

**Figure S7:** Extended data of **Main Figure 3B**, methods according to main manuscript. VirusFISH on MSI biofilms shows an increase in the infection frequency caused by Altivir_1_MSI.

**Figure S8:** Extended data of **Main Figure 3C**, methods according to main manuscript. VirusFISH on MSI biofilms shows that the vast majority of the host cells are infected by Altivir_1_MSI.

**Figure S9:** Extended data of **Main Figure 3D**, methods according to main manuscript. VirusFISH on MSI biofilms shows cell lysis caused by Altivir_1_MSI and the enrichment of filamentous microbes along with the cell debris.

**Figure S10:** Extended data of **Main Figure 4**. Correlation of the 16S rRNA gene relative abundances with the virus-host ratio showing no linear relationship among the most abundant bacterial taxa in individual MSI biofilm flocks.


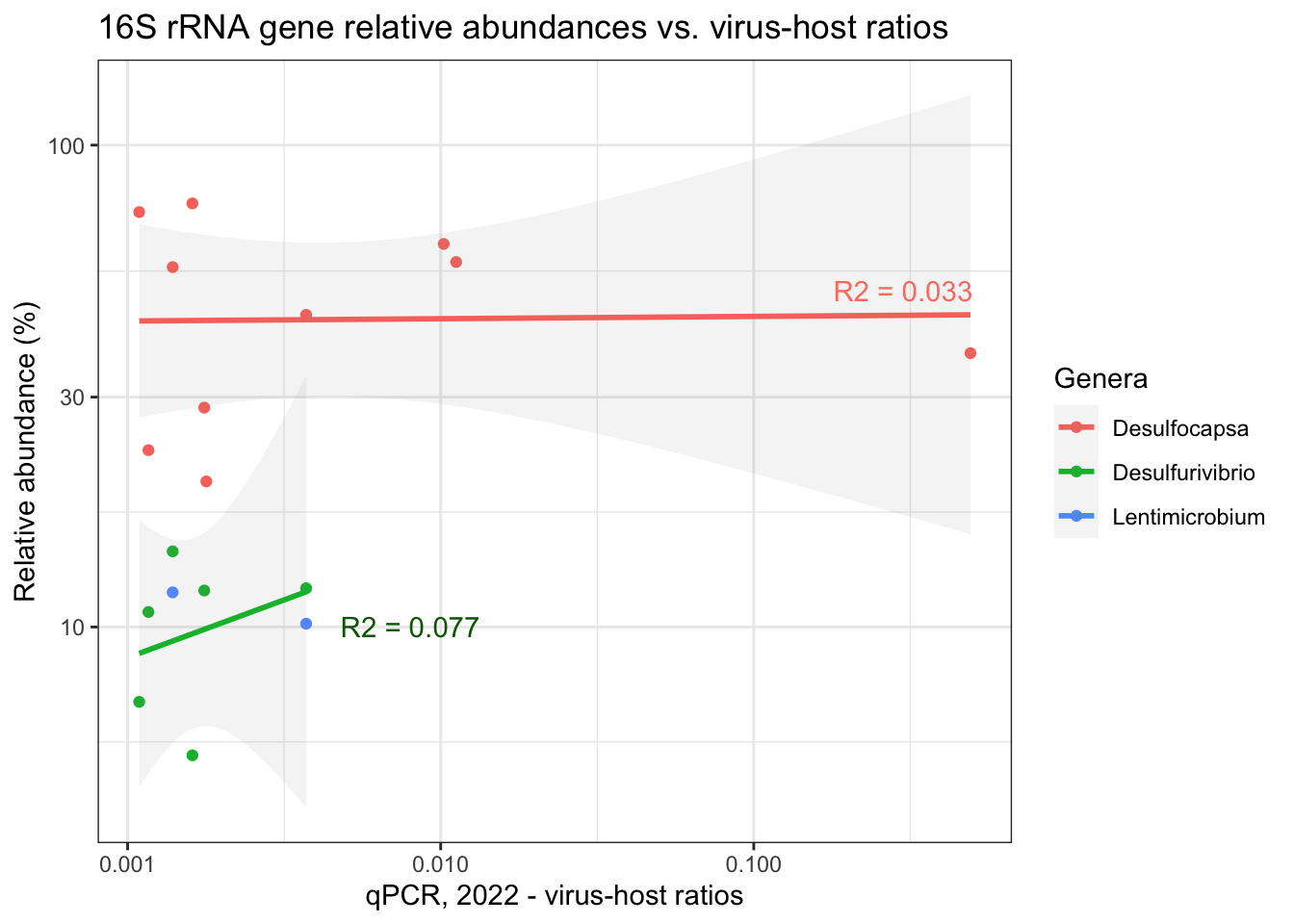


Supplementary References

1. Rahlff, J.; Turzynski, V.; Esser, S.P.; Monsees, I.; Bornemann, T.L.V.; Figueroa-Gonzalez, P.A.; Schulz, F.; Woyke, T.; Klingl, A.; Moraru, C.; et al. Lytic Archaeal Viruses Infect Abundant Primary Producers in Earth’s Crust. *Nat Commun* **2021**, *12*, 4642, doi:10.1038/s41467-021-24803-4.
