# Supplementary figures and images for: Virus-Host Dynamics in Archaeal Groundwater Biofilms and the Associated Bacterial Community Composition
Source: Viruses. 2023 Mar 31;15(4):910. doi: 10.3390/v15040910 (PMC10143303; doi:10.3390/v15040910)

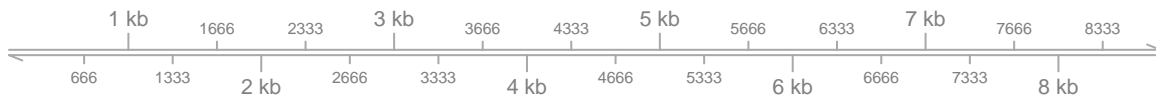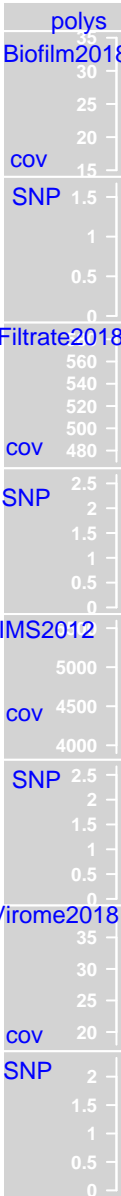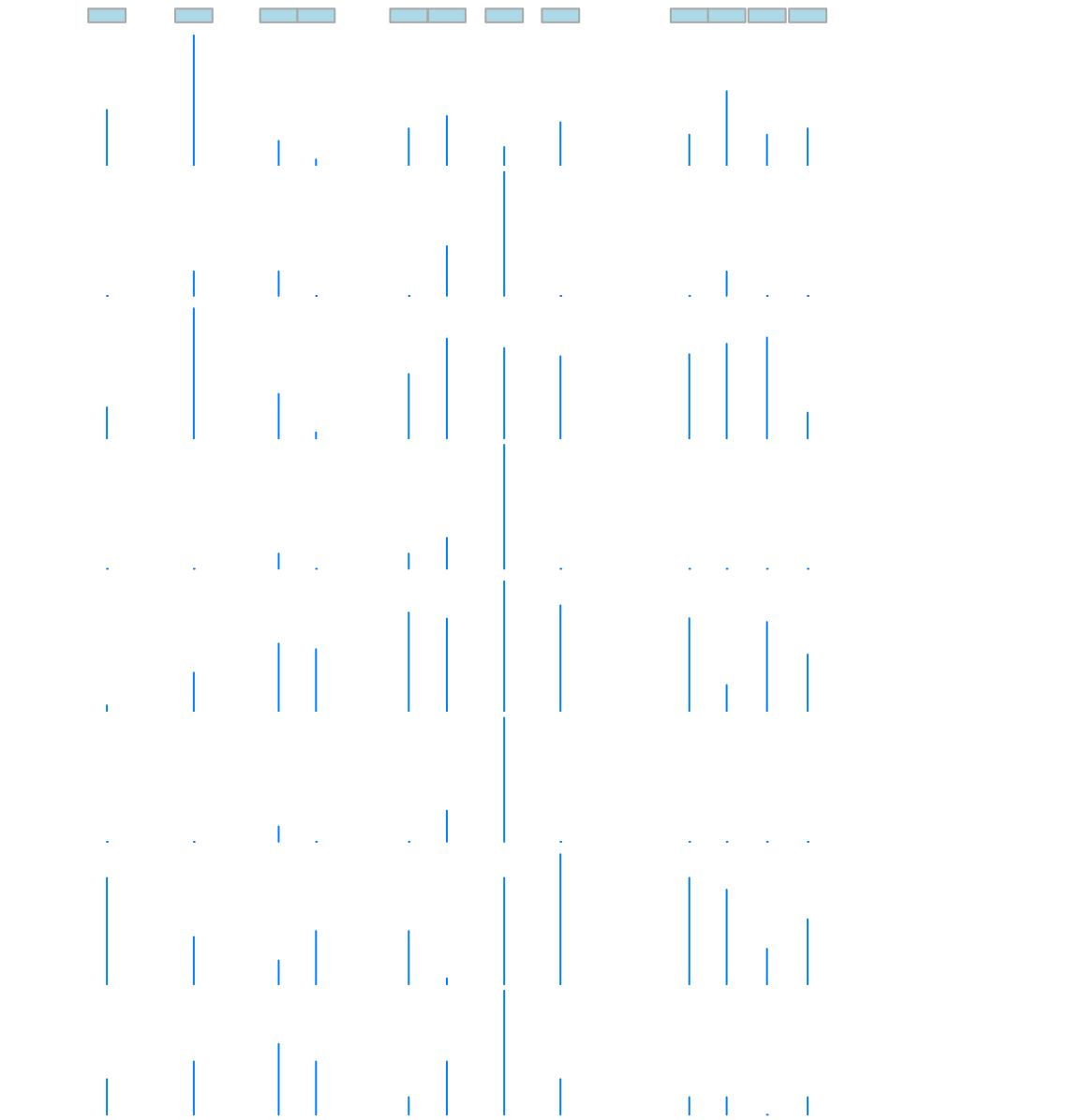

Supplement: Supplementary file 1 [file viruses-15-00910-s001.zip › Supplementary_Turzynski_et_al_2023/Supplementary_Information_2_Plot_polys_Altivir_1.PDF]
