# Supplementary material for: Virus-Host Dynamics in Archaeal Groundwater Biofilms and the Associated Bacterial Community Composition
Source: Viruses. 2023 Mar 31;15(4):910. doi: 10.3390/v15040910 (PMC10143303; doi:10.3390/v15040910)
Supplement: Supplementary file 1 [file Supplementary_Information_1_Plot_Polys_Altiarchaeum.PDF › Supplementary_Turzynski_et_al_2023/Supplementary_Information_1_Plot_Polys_Altiarchaeum.PDF]

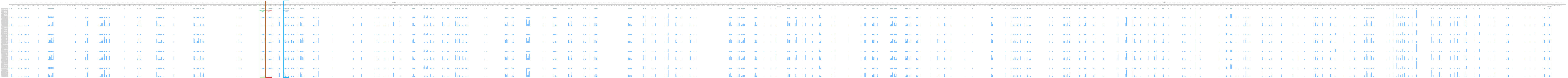
polynucleotide set 1
polynucleotide set 2
polynucleotide set 3
500 kb
